# Poem—The Royal Hand

**Published:** 1889-04-27

**Authors:** 


### The Royal Hand.

In the days when faith was simple,
Love obeyed and asked no word,
When the monarch reigned unquestioned,
As appointed by the Lord,
Then a loyal superstition
Gave him lordship o'er disease,
Thought the pressure of his fingers
To the suffering could give ease.

Were the monarch saint or sinner,
Were he cowardly or brave,
Loved or hated, feared or honoured,
He was king, and he could save;
And upon his passing footsteps
Pressed the sick folk, young and old,
Seeking but to touch his garment,
As they pressed on Christ of old.

Now the old belief has vanished,
No one trusts in mystic power;
Princes' hands have wealth and honour,
But not life to give as dower.
Yet held out in help and blessing
To the suffering of the land,
Is there not a power remaining
In the outstretched royal hand?

Where it points to need or sorrow,
It can lead the people's eyes
To the suffering, oft unheeded,
That around their homesteads lies;
It can mark the path of duty,
It can guide and index be,
To the generous hearts and noble,
To a nation's charity.

This is privilege of kingship,
Thus a people's soul to lead
Unto loving thoughts and tender,
Unto high and generous deed;
This, to be their guide and captain
In the work of doing good,
This, to be the link that binds them
In the chain of brotherhood.

True, the task is not a light one;
Heart and brain must guide the hand,
Endless is the path of duty,
Life is hard to understand.
Every day will bring new labours,
Every day will bring new cares,
All the good is still imperfect,
Wheat is still choked up with tares.

Nor can every prince fulfil it—
Crown nor sceptre make the king,
But the royal soul that striveth
For the right in every thing.
This alone is true and noble,
This is kingship's seal and sign,
And to govern as God's servant
Is to rule by right divine.

